# NLRP3-mediated pyroptosis aggravates pressure overload-induced cardiac hypertrophy, fibrosis, and dysfunction in mice: cardioprotective role of irisin

**DOI:** 10.1038/s41420-021-00434-y

**Published:** 2021-03-15

**Authors:** Rongchuan Yue, Zaiyong Zheng, Yu Luo, Xiaobo Wang, Mingming Lv, Dan Qin, Qingqing Tan, Yulong Zhang, Tao Wang, Houxiang Hu

**Affiliations:** 1 Department of Cardiology, Affiliated Hospital of North Sichuan Medical College, 637000 Nanchong, P.R. China; 2 CSSD, Affiliated Hospital of North Sichuan Medical College, 637000 Nanchong, P.R. China; 3 Ophthalmology Department, Affiliated Hospital of North Sichuan Medical College, 637000 Nanchong, P.R. China; 4 Anesthesiology Department, Affiliated Hospital of North Sichuan Medical College, 637000 Nanchong, P.R. China; 5 Department of Respiratory, Affiliated Hospital of North Sichuan Medical College, 637000 Nanchong, P.R. China

**Keywords:** Cardiovascular diseases, Clinical pharmacology

## Abstract

The exact mechanism of myocardial hypertrophy has not been completely elucidated. NOD-like receptor protein 3 (NLRP3) and the pyroptotic cascade play a critical role in cardiac hypertrophy and inflammation. The myokine irisin can inhibit NLRP3 activation, although its exact mechanism of action is unknown. In this study, we induced cardiac hypertrophy in a mouse model via aortic constriction (TAC) to further explore the pathological role of NLRP3 inflammasome-mediated pyroptosis and the potential therapeutic effects of irisin. Cardiac hypertrophy significantly increased the percentage of apoptotic cells and upregulated IL-1β, cleaved caspase-1, and GSDMD-N that lie downstream of the NLRP3 inflammasome. Subsequently, irisin was co-administered to the TAC mice or angiotensin II (Ang-II)-treated cardiomyocytes to observe whether it could attenuate pyroptosis and cardiac hypertrophy. We established a direct association between pyroptosis and cardiac hypertrophy and found that pharmacological or genetic inhibition of NLRP3 attenuated cardiac hypertrophy. Furthermore, ectopic overexpression of NLRP3 abrogated the cardioprotective effects of irisin. To summarize, pyroptosis is a pathological factor in cardiac hypertrophy, and irisin is a promising therapeutic agent that inhibits NLRP3-mediated pyroptosis of cardiomyocytes.

## Introduction

Heart failure is a chronic condition^1^ that results from cardiac remodeling due to heightened damage or workload, heart damage due to pressure or volume overload, myocardial infarction, inflammatory heart muscle disorder, or idiopathic dilated cardiomyopathy^2,3^. Pathological hypertrophy of the myocardium is a critical risk factor of heart failure^4^ and frequently associated with adverse events despite treatment options^5,6^. Therefore, it is necessary to elucidate the pathophysiological mechanisms underlying cardiac hypertrophy and develop novel therapeutic strategies.

Inflammasomes are pattern-recognition receptors that mediate the inflammatory response against pathogens and other stimuli. The NOD-like receptor protein 3 (NLRP3) inflammasome consists of NLRP3, caspase-1 (cysteinyl aspartate-specific proteinase-1), and ASC (apoptosis-associated speck-like protein). Following activation, NLRP3 recruits and cleaves pro-caspase-1, which triggers the pyroptotic cascade. The NLRP3 inflammasome is activated during several cardiac disorders, such as myocardial infarction, aortic valve diseases, myocarditis, ischemia/reperfusion injury, hypertension, and atherosclerosis^7^, which is consistent with the increased inflammation observed in stress-stimulated cardiac remodeling^8,9^.

Pyroptosis is a pro-inflammatory type of programmed cell death that depends on caspase-1 or caspase-11 stimulation^10,11^ and is morphologically and biochemically similar to necrosis and apoptosis. Unlike the other forms of programmed cell death, however, pyroptosis culminates in the secretion of pro-inflammatory cytokines^12^ and is associated with increased membrane porosity, cellular expansion, and DNA damage^13^. A recent study showed that NLRP3 inflammasome-mediated pyroptosis is a pathological factor underlying cardiovascular disease^14^, although its involvement in cardiac hypertrophy is unknown.

Irisin is a PGC1-α-dependent myokine derived from the extracellular regions of fibronectin type III domain-containing protein 5 (FNDC5) and converts white adipose tissue to brown adipose tissue^15^. It also mitigates nondiabetic obesity and T2DM symptoms by inhibiting inflammation^16,17^. Furthermore, recent studies have implicated a functional role of irisin in cardiovascular diseases, such as atherosclerosis^18^, myocardial ischemia/reperfusion damage, and hypertension^19,20^. It remains to be elucidated whether irisin can ameliorate cardiac hypertrophy and heart failure by inhibiting NLRP3-mediated pyroptosis. To this end, we established in vivo and in vitro models of cardiac hypertrophy and analyzed the effects of irisin on pressure overload-stimulated cardiac hypertrophy and NLRP3-mediated pyroptosis.

## Results

### Pressure overload causes pyroptosis in the heart tissue and cultured cardiomyocytes

**Fig. 1:**
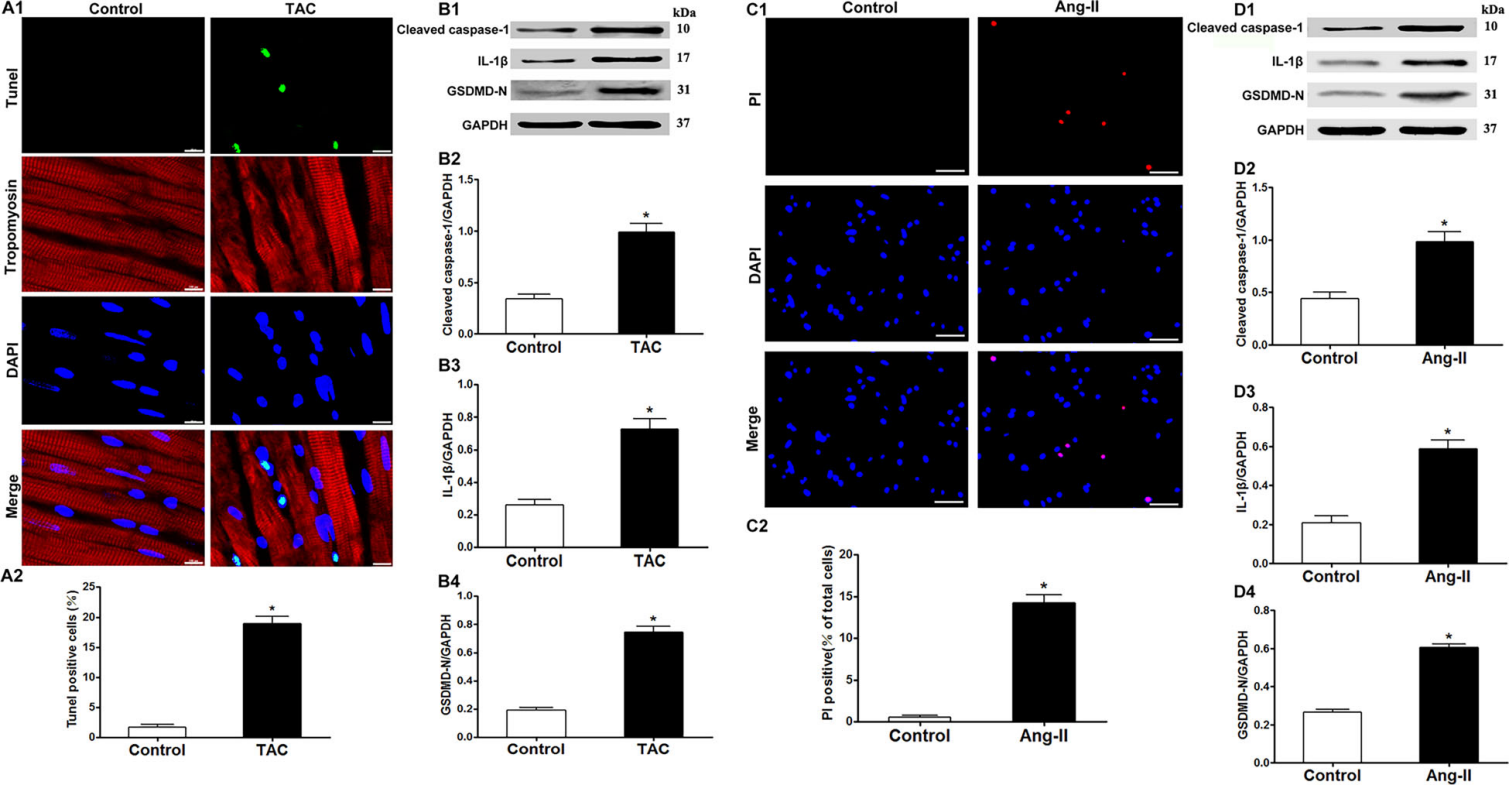
TAC and Ang-II, respectively, induce pyroptosis in myocardial tissue and cultured cardiomyocytes. **A1** Representative images showing TUNEL + pyroptotic cardiomyocytes. Red—anti-tropomyosin, blue nuclei—DAPI, green nuclei—TUNEL. Scale bars: 100 μm. **A2** Percentage of TUNEL-positive cells in the indicated groups. **B** Immunoblots showing the levels of cleaved caspase-1 (**B1, B2**), IL-1β (**B1**, **B3**), and GSDMD-N (**B1**, **B4**) in the indicated groups. **C1** Representative images showing PI-stained pyroptotic cardiomyocytes with/out Ang-II treatment. Blue nuclei—DAPI, red nuclei—PI. Scale bars: 200 μm. **C2** Percentage of PI-positive cells in the indicated groups. **D** Immunoblots showing levels of cleaved caspase-1 (**D1**, **D2**), IL-1β (**D1, D3**), and GSDMD-N (**D1**, **D4**) in the suitably treated cardiomyocytes. Results are mean ± SEM, *n* = 6. *P < 0.01 compared to control.

A cardinal feature of pyroptosis is rapid membrane disintegration, which can be assessed with membrane-impermeable dyes, such as fluorescein-dUTP and PI^21,22^. As shown in Fig. 1A, the heart tissues from mice with cardiac hypertrophy had a significantly higher proportion of TUNEL + apoptotic cells compared to that from sham-operated controls. Consistent with this, the levels of cleaved caspase-1 (Fig. 1B1, B2) and IL-1β (Fig. 1B1, B3) were also upregulated in the myocardium of the TAC group relative to the control group. Furthermore, the specific pyroptosis marker GSDMD-N was also upregulated in the TAC group compared to the controls (Fig. 1B1, B4). Consistent with the in vivo results, the Ang-II-treated cardiomyocytes also showed significantly higher pyroptosis rates compared to the untreated cells, as indicated by the increased percentage of PI-positive cells (Fig. 1C), and upregulation of cleaved caspase-1 (Fig. 1D1, D2), IL-1β (Fig. 1D1, D3), and GSDMD-N (Fig. 1D1, D4) in the former. Taken together, pressure overload on cardiomyocytes induces pyroptosis.

### Caspase-1 inhibition downregulates pressure overload-induced pyroptosis

**Fig. 2:**
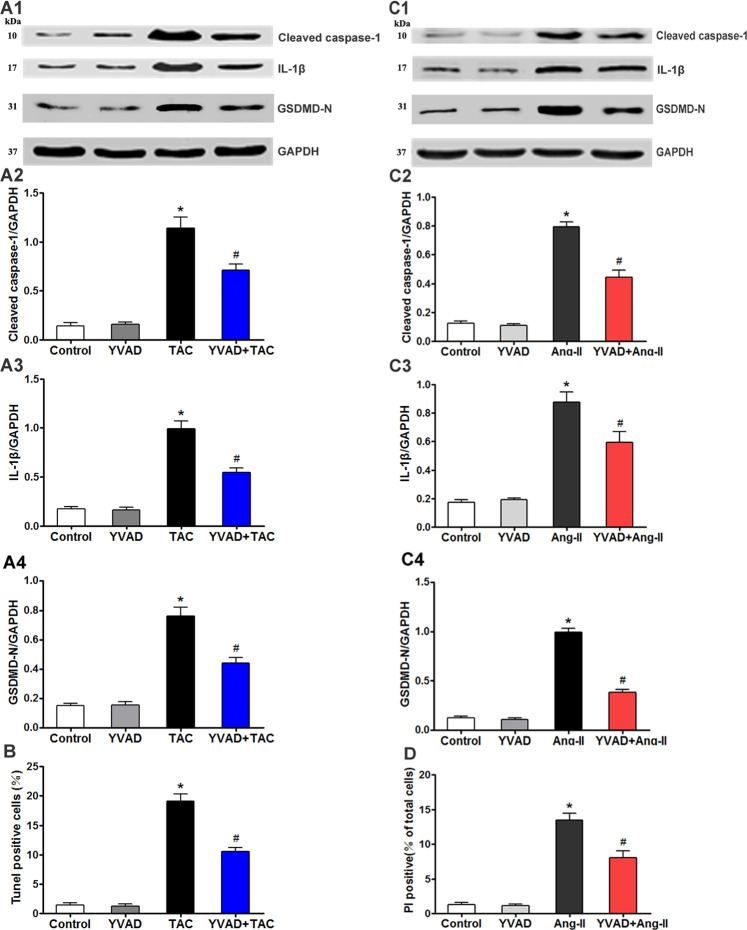
Caspase-1 inhibition downregulates overload-induced pyroptosis. **A** Immunoblots showing levels of cleaved caspase-1 (**A1**, **A2**), IL-1β (**A1**, **A3**), and GSDMD-N (**A1**, **A4**) in the indicated groups. **B** Percentage of TUNEL-positive cells in the indicated groups. **C** Immunoblots showing levels of cleaved caspase-1 (**C1**, **C2**), IL-1β (**C1**, **C3**), and GSDMD-N (**C1**, **C4**) in the suitably treated cardiomyocytes. **D** Percentage of PI-positive cells in the indicated groups. Results are mean ± SEM, *n* = 6. *P < 0.01 compared to control, ^#^*P* < 0.01 compared to TAC group or Ang-II group. YVAD (AC-YVAD-CMK, caspase-1 inhibitor).

To establish the potential correlation between pyroptosis and cardiac pressure overload, we analyzed the effects of caspase-1 inhibition on pressure overload-stimulated pyroptosis. The sham-operated and TAC mice were additionally treated with the caspase-1 inhibitor AC-YVAD-CMK (YVAD), which not only mitigated the high levels of cleaved caspase-1 (Fig. 2A1, A2), IL-1β (Fig. 2A1, A3), and GSDMD-N (Fig. 2A1, A4) induced by TAC but also decreased the percentage of TUNEL-positive cells (Fig. 2B). Likewise, cardiomyocytes co-treated with YVAD and Ang-II expressed lower levels of cleaved caspase-1 (Fig. 2C1, C2), IL-1β (Fig. 2C1, C3), and GSDMD-N (Fig. 2C1, C4) compared to the Ang-II-treated cells. In addition, YVAD also decreased the percentage of PI-positive cells in the presence of Ang II (Fig. 2D). Thus, YVAD mitigates pressure overload-induced pyroptosis in cardiomyocytes, indicating that activation of pyroptosis is directly related to pressure overload.

### Caspase-1 inhibition attenuates overload-stimulated cardiac hypertrophy

**Fig. 3:**
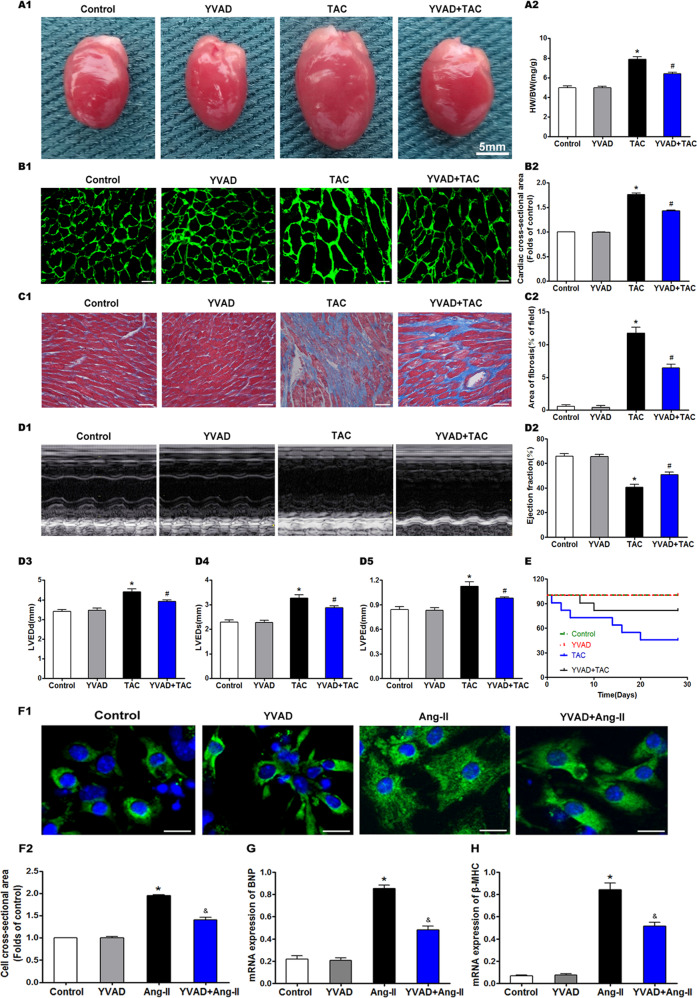
Caspase-1 inhibition attenuates overload-stimulated cardiac hypertrophy. **A1** Representative pictures showing mouse hearts in the indicated groups. **A2** Proportion of heart to body weight in the indicated groups. **B1** Representative images showing the cross-sectional area of cardiomyocytes of the left ventricle in the indicated groups. Heart tissues were immune-stained with WGA. Green—anti-WGA. One hundred cells were randomly selected from five sections of each heart. Scale bars: 20 μm. **B2** Quantification of the cross-sectional area of cardiomyocytes of the left ventricle in the indicated groups. **C1** Representative images showing the myocardial fibrosis, assessed using Masson trichrome staining in the indicated groups. Blue areas represent fibrotic staining. Fibrosis was quantified by five whole LV sections for each heart. Scale bars: 50 μm. **C2** Quantification of the myocardial fibrosis in the indicated groups. The cardiac structure and function of mice were assessed by echocardiography 4 weeks after TAC. **D1** Representative images of M-mode echocardiography for mouse hearts. Quantitative analysis of left ventricular EF (**D2**), LVEDd (**D3**), LVEDs (**D4**), and LVPWd (**D5**). **E** Survival curves of mice using Kaplan and Meier. Characteristic images (**F1**) and measurement (**F2**) of the cell surface area of cardiomyocytes. Cardiomyocytes were stained with cardiomyocyte marker α-actinin (green fluorescence). Nuclei were marked with DAPI (blue fluorescence), Scale bars: 20 μm. In total, 100 cells were selected on random and assessed for surface area for each cohort from six independent trials. Real-time PCR of mRNA expression of cardiac hypertrophy markers BNP (**G**) and β-MHC (**H**). Results are presented as the mean ± SEM, *n* = 6. ^∗^*P* < 0.01 compared to control, ^#^*P* < 0.01 compared to TAC group, ^&^*P* < 0.01 compared to Ang-II group.

To clarify the relationship between pyroptosis and cardiac hypertrophy, we analyzed the effect of YVAD on overload-stimulated cardiac hypertrophy, fibrosis, and heart failure. TAC significantly increased the cardiac mass as well as the proportion of heart weight (HW) to body weight (BW), suggesting that pressure overload led to cardiac hypertrophy (Fig. 3A). While YVAD significantly repressed the TAC-induced increase in HW/BW ratio, it had no effect on the healthy heart. Furthermore, YVAD also inhibited the increase in mean cardiomyocyte surface area following TAC (Fig. 3B). Pathological myocardial hypertrophy often leads to myocardial fibrosis, which is one of the mechanisms underlying heart failure. The mice hearts showed significant interstitial fibrosis and collagen accumulation 4 weeks after TAC (Fig. 3C). YVAD had no influence on the sham-operated hearts but significantly reduced the fibrotic area in the myocardium after TAC (Fig. 3C). Furthermore, the echocardiographic assessment indicated that TAC decreased the EF, and while YVAD significantly alleviated cardiac dysfunction, it had no major effect on the cardiac function of the sham-operated mice (Fig. 3D2). TAC mice showed a substantial upregulation in LVEDd (Fig. 3D3) and LVEDs (Fig. 3D4), which were significantly reversed by YVAD. The LVPWd of the hearts had thickened 4 weeks after TAC and was controlled by YVAD (Fig. 3D5). Thus, YVAD prevented TAC-induced cardiac dysfunction and alteration in ventricular structure. In addition, the survival rate of mice that underwent TAC was 63.6% at 2 weeks post surgery and dropped further to 45.5% at 4 weeks, which was consistent with the cardiac dysfunction induced by TAC. However, YVAD therapy sustained the survival rate at 81.8% at both 2 and 4 weeks after TAC (Fig. 3E). YVAD also significantly decreased the surface area of the Ang-II-treated cardiomyocytes in vitro (Fig. 3F1, F2), which correlated with the downregulation of hypertrophy-related markers, such as brain natriuretic peptide (BNP) (Fig. 3G) and β-myosin heavy chain (β-MHC) (Fig. 3H). Taken together, the activation of pyroptosis is correlated with cardiac hypertrophy, and its inhibition can attenuate overload-stimulated cardiac hypertrophy and heart failure.

### Irisin attenuates pressure overload-induced pyroptosis and cardiac hypertrophy

**Fig. 4:**
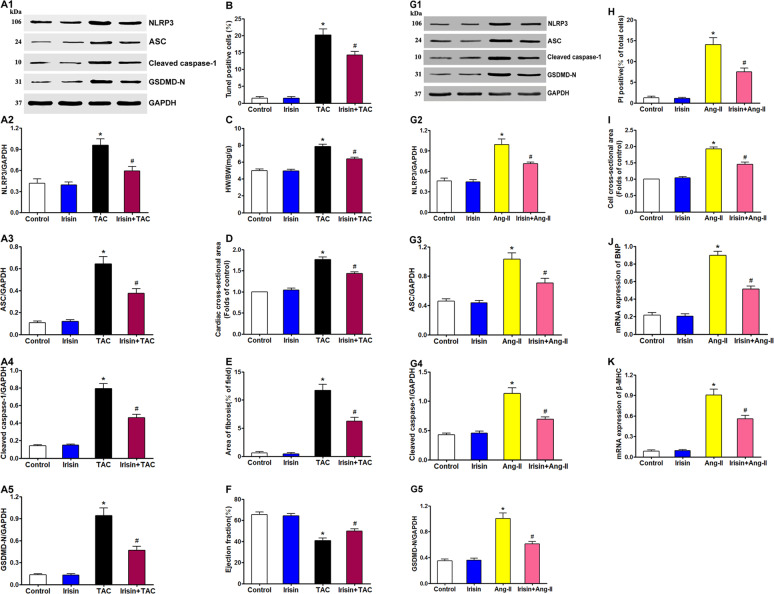
Irisin inhibits overload-stimulated pyroptosis and ameliorates cardiac hypertrophy and cardiac function. **A** Immunoblots showing protein levels of NLRP3 (**A1**, **A2**), ASC (**A1**, **A3**), cleaved caspase-1 (**A1**, **A4**), and GSDMD-N (**A1**, **A5**) in the indicated groups. **B** Percentage of TUNEL-positive cells in the indicated groups. **C** Proportion of heart to body weight in the indicated groups. **D** Quantification of the cross-sectional area of cardiomyocytes of the left ventricle in the indicated groups. **E** Quantification of the myocardial fibrosis in the indicated groups. **F** Quantitative analysis of left ventricular EF in the indicated groups. **G** Immunoblots showing protein levels of NLRP3 (**G1**, **G2**), ASC (**G1**, **G3**), cleaved caspase-1 (**G1**, **G4**), and GSDMD-N (**G1**, **G5**) in the suitably treated cardiomyocytes. **H** Percentage of PI-positive cells in the suitably treated cardiomyocytes. **I** Measurement of the cell surface area of cardiomyocytes in the indicated groups. mRNA expression of cardiac hypertrophy markers BNP (**J**) and β-MHC (**K**) in the suitably treated cardiomyocytes. Results are presented as the mean ± SEM, *n* = 6. ^∗^*P* < 0.01 compared to control, ^#^*P* < 0.01 compared to TAC group or Ang-II group.

To determine whether the NLRP3 inflammasome is stimulated by cardiac overload, the sham-operated and TAC-modeled mice were treated with irisin. TAC-induced increase in NLRP3 (Fig. 4A1, A2), ASC (Fig. 4A1, A3), cleaved caspase-1 (Fig. 4A1, A4), and GSDMD-N (Fig. 4A1, A5) protein levels in the cardiac tissues were significantly reduced by irisin treatment. Co-treatment with irisin also decreased the percentage of TUNEL-positive cells compared with the untreated TAC group (Fig. 4B). Consistent with this, irisin downregulated the Ang-II-induced increase in NLRP3 (Fig. 4G1, G2), IL-1β (Fig. 4G1, G3), cleaved caspase-1 (Fig. 4G1, G4), and GSDMD-N (Fig. 4G1, G5) in the cardiomyocytes, and also decreased the percentage of PI-positive cells (Fig. 4H). These results indicate that the NLRP3 inflammasome is directly associated with pressure overload-induced pyroptosis in the cardiomyocytes. The enhanced HW/BW ratio in TAC mice was also significantly suppressed by irisin, which did not affect the healthy heart (Fig. 4C). Furthermore, irisin prevented the TAC-induced increase in the mean surface area of cardiomyocytes (Fig. 4D) and significantly reduced the myocardial fibrotic area (Fig. 4E). As shown in Fig. 4F, irisin significantly alleviated cardiac dysfunction caused by TAC but had no drastic influence on the cardiac function of the sham-operated mice. In addition, the cardiomyocytes co-treated with irisin and Ang II had lower surface area and reduced expression of BNP (Fig. 4J) and β-MHC (Fig. 4K) compared to those stimulated with Ang-II. Taken together, irisin mitigated pressure overload-induced pyroptosis, as well as cardiac hypertrophy and fibrosis, by targeting the NLRP3 inflammasome.

### Inhibition of NLRP3 inflammasome alleviates pyroptosis and overload-stimulated cardiac hypertrophy

**Fig. 5:**
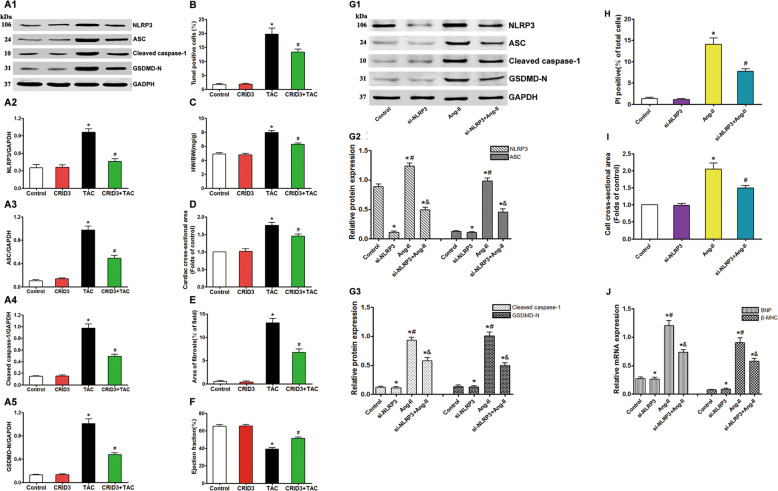
NLRP3 inhibitor CRID3 or knockdown NLRP3 with si-RNA decreased pyroptosis and attenuated cardiac hypertrophy induced by TAC or Ang-II. **A** Immunoblots showing the protein levels of NLRP3 (**A1**, **A2**), ASC (**A1**, **A3**), cleaved caspase-1 (**A1**, **A4**), and GSDMD-N (**A1**, **A5**) in the cardiac tissue of indicated groups. **B** Percentage of TUNEL-positive cells in the indicated groups. **C** Proportion of heart to body weight in the indicated groups. **D** Quantification of the cross-sectional area of cardiomyocytes of the left ventricle in the indicated groups. **E** Quantification of the myocardial fibrosis in the indicated groups. **F** Quantitative analysis of left ventricular EF in the indicated groups. **G** Immunoblots showing protein levels of NLRP3 and ASC (**G1**, **G2**), cleaved caspase-1, and GSDMD-N (**G1**, **G3**) in the suitably treated cardiomyocytes. **H** Percentage of PI-positive cells in the suitably treated cardiomyocytes. **I** Measurement of the cell surface area of cardiomyocytes in the indicated groups. **J** mRNA expression of cardiac hypertrophy markers BNP and β-MHC in the suitably treated cardiomyocytes. Results are presented as the mean ± SEM, *n* = 6. ^∗^*P* < 0.01 compared to control, ^#^*P* < 0.01 compared to TAC or Ang-II group. CRID3 (cytokine release inhibitory drug 3, NLRP3 inhibitor) known as CP-456773 or MCC950.

The NLRP3 inflammasome is activated in various inflammatory disorders^23–25^. TAC significantly upregulated the NLRP3 (Fig. 5A1, A2), ASC (Fig. 5A1, A3), cleaved caspase-1 (Fig. 5A1, A4), and GSDMD-N (Fig. 5A1, A5) proteins in the myocardium, and increased the number of TUNEL-positive cells (Fig. 5B). Cytokine release inhibitory drug 3 (CRID3) (200 mg/kg/, dosed twice a day by oral gavage), a selective inhibitor of the NLRP3 inflammasome, reversed the TAC-induced pyroptosis cascade. Consistent with this, siRNA-mediated knockdown of NLRP3 in the Ang-II-treated cardiomyocytes decreased expression levels of NLRP3, ASC (Fig. 5G1, G2), cleaved caspase-1, and GSDMD-N (Fig. 5G1, G3), as well the number of PI-positive cells (Fig. 5H). CRID3 prevented the TAC-induced elevation in HW/BW ratio but did not affect the mass of the normal heart (Fig. 5C). It also reversed the increase in mean cardiomyocyte surface area in the TAC mice (Fig. 5D), significantly decreased the myocardial fibrotic area (Fig. 5E), and normalized the EF (Fig. 5F). NLRP3-knockdown cardiomyocytes treated with Ang II had a lower surface area (Fig. 5I), and decreased levels of BNP and β-MHC (Fig. 5J) compared to the normal Ang-II-stimulated cells. Thus, inhibition of the NLRP3 inflammasome can improve the symptoms of overload-stimulated cardiac hypertrophy and heart failure.

### Overexpression of NLRP3 mitigates the cardioprotective effects of irisin

**Fig. 6:**
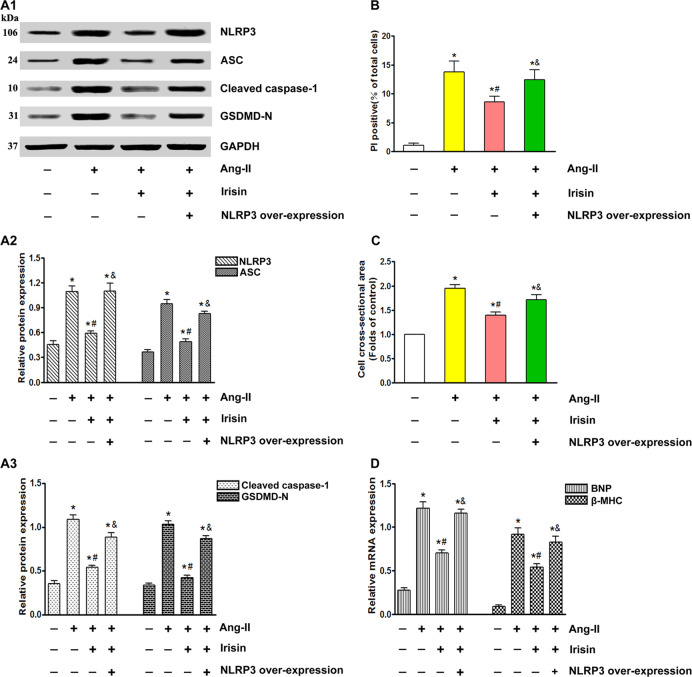
Overexpression of NLRP3 reversed irisin-mediated antipyroptosis and anticardiac hypertrophy effects. **A** Immunoblots showing protein levels of NLRP3 and ASC (**A1**, **A2**), cleaved caspase-1, and GSDMD-N (**A1**, **A3**) in the suitably treated cardiomyocytes. **B** Percentage of PI-positive cells in the suitably treated cardiomyocytes. **C** Measurement of the cell surface area of cardiomyocytes in the indicated groups. **D** mRNA expression of cardiac hypertrophy markers BNP and β-MHC in the suitably treated cardiomyocytes. Results are presented as the mean ± SEM, *n* = 6. ^∗^*P* < 0.01 compared to control, ^#^*P* < 0.01 compared to Ang-II group, ^&^*P* < 0.01 versus Ang-II + Irisin group.

To evaluate the role of exogenous irisin in controlling NLRP3-stimulated inflammation, we treated ectopic NLRP3-overexpressing cardiomyocytes with irisin. The high levels of NLRP3 reversed the irisin-mediated suppression of ASC, cleaved caspase-1, and GSDMD-N (Fig. 6A), and also increased the number of PI-positive cells (Fig. 6B), cardiomyocyte surface area (Fig. 6C), and the BNP and β-MHC levels (Fig. 6D). To summarize, NLRP3 inflammasome-mediated signaling influences the antipyroptotic and antihypertrophic effects of irisin.

## Discussion

We found that pyroptosis is associated with cardiac hypertrophy and the cardioprotective effects of irisin are mediated by inhibiting pyroptosis. Moreover, we also confirmed that the pressure overload-induced pyroptosis is driven by the NLRP3 inflammasome, and inhibiting the pyroptotic cascade alleviates cardiac hypertrophy. Finally, the cardioprotective effects of irisin were abrogated by NLRP3 overexpression, which underscored that NLRP3-mediated pyroptosis is the main target of irisin.

Pyroptosis refers to a form of pro-inflammatory cell death that is contingent on caspase-1 stimulation^26,27^ and is the pathological basis of numerous disorders. For instance, nicotine promotes atherosclerosis by inducing pyroptosis of endothelial cells^28^. In addition, endothelial pyroptosis is also prevalent during endotoxemia-stimulated lung damage^29^. The close relationship between heart disease and pyroptosis has also been demonstrated in previous studies^30–32^. Nevertheless, the molecular mechanisms underlying pyroptosis in cardiac hypertrophy were not clear. We established in vivo and in vitro models of cardiac hypertrophy via TAC and Ang-II treatment, respectively, and observed a significant increase in pyroptotic cells, along with upregulation in IL-1β, cleaved caspase-1, and GSDMD-N. These findings clearly indicated that pyroptosis has a pathological role in cardiac hypertrophy.

Irisin is abundant in the adipose tissues, skeletal muscles, and heart^33,34^. A previous study showed that irisin levels were decreased in cardiac tissues and plasma after TAC, as well as in Ang-II-stimulated cardiomyocytes^35^. Since the cardiac tissues are the main source of irisin^36^, loss of cardiomyocytes may contribute to a reduction in irisin after hypertrophy. There is evidence that exogenous irisin can reverse NLRP3-stimulated inflammation^37^, indicating that exogenous supplementation of recombinant irisin may partially reverse cardiac hypertrophy. We found that irisin normalized the aberrantly high levels of IL-1β, cleaved caspase-1, and GSDMD-N after TAC or Ang-II treatment. In the mouse model, irisin mitigated TAC-induced cardiac hypertrophy and fibrosis, as indicated by reduced HW/BW, smaller cardiomyocytes, and improved echocardiography parameters (EF). Thus, irisin can ameliorate heart failure and cardiac hypertrophy by inhibiting pyroptosis.

Caspase-1 activation by the inflammasome complex leads to the cleavage and secretion of pro-inflammatory IL-1β^38^. Inflammasomes can trigger both caspase-1-dependent and -independent pyroptosis following tissue injury^39–42^. Our findings indicated that NLRP3/caspase-1-dependent pyroptosis has a pathological role in myocardial hypertrophy. Targeted inhibition of NLRP3 by CRID3 and gene silencing blocked caspase-1-dependent pyroptosis in cardiomyocytes and alleviated the signs of cardiac hypertrophy. Furthermore, irisin inhibited NLRP3-mediated pyroptosis, and its cardioprotective effects were abrogated by overexpression of NLRP3.

To summarize, NLRP3-mediated pyroptosis has a vital function in cardiac hypertrophy, and irisin can mitigate cardiac hypertrophy and heart failure by inhibiting the pyroptosis cascade.

## Materials and methods

### Mouse model of cardiac hypertrophy

Cardiac hypertrophy was established in 4-month-old male C57BL/6J mice using transverse aortic constriction (TAC)^43^. The mice were anesthetized and placed in a supine position, and a midline cervical incision was made to expose the trachea. After opening the chest cavity, the transverse aorta was sutured using 7–0 nylon thread, connected using a blunt 27-gauge needle, and then detached. The sham-operated controls underwent the same procedure without aortic ligation. The chest was then sutured, and the animals were ventilated until they could breathe on their own. The mice were intravenously injected with PBS (control) or recombinant irisin (2 μg/kg/week, Phoenix Pharmaceuticals, Inc., USA)^44^ at 0, 1, 2, and 3 weeks after TAC, and euthanized after 4 weeks. The cardiac tissues were resected for further analysis. All experimental procedures were conducted as per the guidelines for the Animal Care and Use Committee of North Sichuan Medical College, China, and approved by the Medical Ethics Committee.

### In vitro model of cardiomyocyte hypertrophy

Primary cardiomyocytes were obtained from 1- to 3-day-old neonatal mice as previously described^45^, and cultured for 12 h in serum-free DMEM. According to published protocols^44^, the cardiomyocytes were treated with PBS or Ang-II (1 ng/ml) with/out irisin (100 ng/ml) for 0, 0.5, 1, 24, and 48 h. The serum-free medium containing Ang II was changed every 24 h, and the cells were harvested for further analysis.

### Echocardiography

The mice were anesthetized with a combination of oxygen (2 L/min) and isoflurane (2%) and subjected to transthoracic echocardiography at stipulated time points using GE vivid 8-dimension ultrasound. The heart was observed on the short axis among two papillary muscles, and every dimension was acquired using M-mode by taking the mean of three successive heartbeats. The EF% (LV ejection fraction), LVEDs (left ventricular end-systolic diameter), LVEDd (left ventricular end-diastolic dimension), and LVPWd (left ventricular diastolic posterior wall thickness) were measured by the same technician, and the average of six consecutive heart cycles was calculated.

### Histology and immunohistochemistry

The hearts of the model and sham-operated mice were resected 4 weeks post TAC, weighed, perfused with PBS, fixed with 4% (w/v) paraformaldehyde, and paraffin-embedded or snap-frozen in liquid nitrogen. The paraffin-embedded tissues were cut into 4-μm-thick sections. After blocking with goat serum at room temperature for 30 min, the sections were immunostained with anti-wheat germ agglutinin (WGA) antibody to assess the cross-sectional area of the cardiomyocytes^46^. The degree of fibrosis was assessed by Masson trichrome staining of the left ventricular wall sections. Five random high-powered fields were examined per section in a blinded manner. Images were analyzed using the ImageJ program.

### Immunocytochemistry

Cardiomyocytes were cultured on coverslips in vitro as appropriate and fixed in 4% paraformaldehyde at room temperature for 20 min. After permeabilizing in 0.1% Triton X-100 for 10 min, the cells were blocked with 3% BSA for 30 min and then incubated with the anti-α-actinin antibody (1:100, Abcam). The cells were then probed with Alexa Fluor 647-conjugated secondary antibody at room temperature for 60 min, and the nuclei were counterstained with DAPI. The stained cells were observed under a fluorescence microscope (Nikon, Japan), and the surface area of each cardiomyocyte was quantified using ImageJ. Five independent zones were selected per slide and the area was quantified in a blinded manner and normalized to the control group.

### TUNEL assay

TUNEL assay was performed as described previously^47^ using the in situ cell death detection system (Roche, USA). The cardiomyocytes were stained using the sarcomeric tropomyosin antibody and the nuclei were counterstained with DAPI. The cells were observed under a Nikon fluorescence microscope (Nikon, Tokyo, Japan), and the average fluorescence intensity was assessed with the ImageJ software. TUNEL-positive nuclei were counted in 15 nonoverlapping regions, and the percentage was calculated relative to the total number of cells per field. The experiment was repeated six times in a blinded manner.

### Propidium iodide (PI) staining

The suitably treated cells were washed with PBS and stained with DAPI (5 ml) and PI (5 ml) for 20 min in the dark at 37 °C. The PI-positive cells were counted per 200 cells in three random fields under a fluorescence microscope (Nikon, Tokyo, Japan), and the average fluorescence intensity was assessed with the ImageJ software.

### Real-time RT-PCR

RNA was extracted from the cardiac tissues using the RNAiso plus kit (TAKARA). The transcript levels of brain natriuretic peptide (BNP) and β-myosin heavy chain (β-MHC) were analyzed by RT-PCR using the TOYOBO kit and the iQ5 Real-Time PCR Detection System (Bio-Rad, Hercules, CA) as per instructions. The SYBR1 premix Ex TaqTM II was used according to the recommended guidelines (TaKaRa Biotechnology Co. Ltd., Tokyo, Japan), and GAPDH was the internal standard. The melting curves and values were assessed with the Bio-RAD software. The relative mRNA level was quantified using the comparative cycle threshold method. Each sample was analyzed at least thrice.

### Western blotting

Western blotting was conducted as described^48^ using antibodies against ASC (1:1000, Abcam, ab151700), GSDMD-N (1:200, Abcam, ab215203), NLRP3 (1:200, Abcam, ab270449), IL-1β (1:1000, Abcam, ab200478), cleaved caspase-1 (1:200, Abcam, ab238972), and the internal control GAPDH (1:1000, Abcam, ab8245). The protein bands were quantified using the Odyssey color infrared laser scanning imager (LI-COR, Lincoln, NE).

### Statistical analyses

All results were presented as mean ± SEM of five independent experiments. GraphPad Prism 5.0 software (San Diego, CA, USA) was used for statistical analysis. One-way factorial ANOVA was used to compare multiple groups, and the Student’s *t* test for pairwise comparison. The Kaplan–Meier method was used for survival analysis. *P* < 0.05 was considered statistical significance.

## Data Availability

The data used to support the findings of this study are included in the paper.
