# Correction: NLRP3-mediated pyroptosis aggravates pressure overload-induced cardiac hypertrophy, fibrosis, and dysfunction in mice: cardioprotective role of irisin

**DOI:** 10.1038/s41420-026-03177-w

**Published:** 2026-07-15

**Authors:** Rongchuan Yue, Zaiyong Zheng, Yu Luo, Xiaobo Wang, Mingming Lv, Dan Qin, Qingqing Tan, Yulong Zhang, Tao Wang, Houxiang Hu

**Affiliations:** 1 Department of Cardiology, Affiliated Hospital of North Sichuan Medical College, 637000 Nanchong, P.R. China; 2 CSSD, Affiliated Hospital of North Sichuan Medical College, 637000 Nanchong, P.R. China; 3 Ophthalmology Department, Affiliated Hospital of North Sichuan Medical College, 637000 Nanchong, P.R. China; 4 Anesthesiology Department, Affiliated Hospital of North Sichuan Medical College, 637000 Nanchong, P.R. China; 5 Department of Respiratory, Affiliated Hospital of North Sichuan Medical College, 637000 Nanchong, P.R. China

Correction to: *Cell Death Discovery* 10.1038/s41420-021-00434-y, published online 15 March 2021

During figure assembly and preparation,the wrong image file was inadvertently selected and inserted for Figure lC due to a simple administrative mistake. This error was entirely unintentional and in no way affects the validity of the results or conclusions.

**Figure 1 Original** 
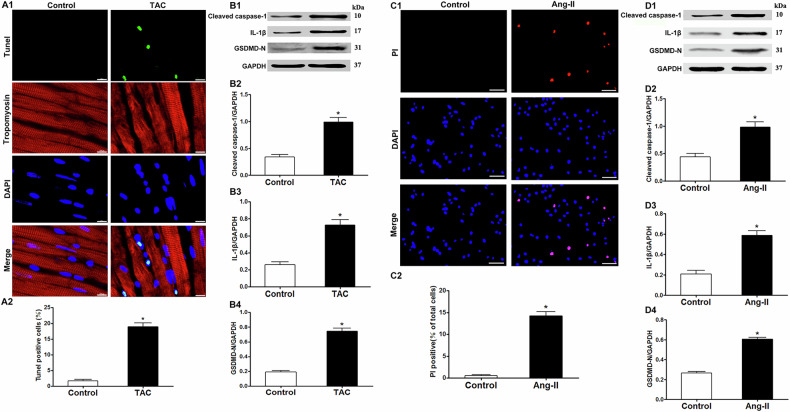


**Figure 1 Amended** 
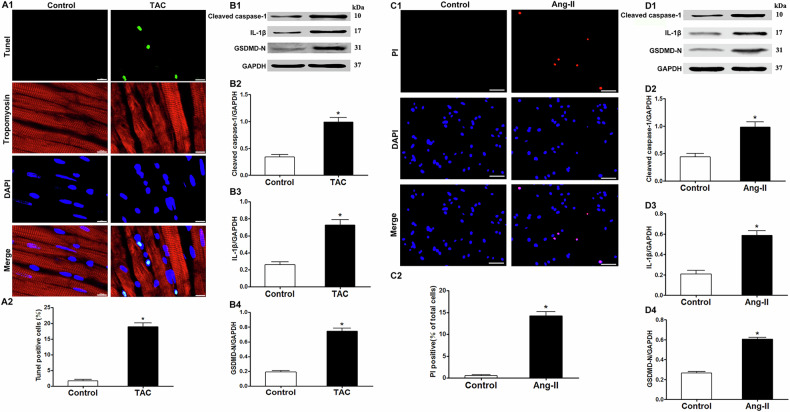


The original article has been corrected.

